# FLT3-TKD in the prognosis of patients with acute myeloid leukemia: A meta-analysis

**DOI:** 10.3389/fonc.2023.1086846

**Published:** 2023-02-17

**Authors:** Shuping Li, Na Li, Yun Chen, Zhihua Zheng, Yao Guo

**Affiliations:** ^1^ Edmond H. Fischer Translational Medical Research Laboratory, Scientific Research Center, The Seventh Affiliated Hospital, Sun Yat-sen University, Shenzhen, Guangdong, China; ^2^ Department of Nephrology, Center of Nephrology, The Seventh Affiliated Hospital, Sun Yat-Sen University, Shenzhen, Guangdong, China

**Keywords:** AML, FLT3-TKD, meta-analysis, metabolism, prognosis

## Abstract

**Background:**

Fms-like tyrosine kinase 3 (FLT3) gene mutations occur in approximately 30% of all patients with acute myeloid leukemia (AML). Internal tandem duplication (ITD) in the juxtamembrane domain and point mutations within the tyrosine kinase domain (TKD) are two distinct types of FLT3 mutations. FLT3-ITD has been determined as an independent poor prognostic factor, but the prognostic impact of potentially metabolically related FLT3-TKD remains controversial. Hence, we performed a meta-analysis to investigate the prognostic significance of FLT3-TKD in patients with AML.

**Methods:**

A systematic retrieval of studies on FLT3-TKD in patients with AML was performed in PubMed, Embase, and Chinese National Knowledge Infrastructure databases on 30 September 2020. Hazard ratio (HR) and its 95% confidence intervals (95% CIs) were used to determine the effect size. Meta-regression model and subgroup analysis were used for heterogeneity analysis. Begg’s and Egger’s tests were performed to detect potential publication bias. The sensitivity analysis was performed to evaluate the stability of findings in meta-analysis.

**Results:**

Twenty prospective cohort studies (n = 10,970) on the prognostic effect of FLT3-TKD in AML were included: 9,744 subjects with FLT3-WT and 1,226 subjects with FLT3-TKD. We found that FLT3-TKD revealed no significant effect on disease-free survival (DFS) (HR = 1.12, 95% CI: 0.90–1.41) and overall survival (OS) (HR = 0.98, 95% CI: 0.76–1.27) in general. However, meta-regressions demonstrated that patient source contributed to the high heterogeneity observed in the prognosis of FLT3-TKD in AML. To be specific, FLT3-TKD represented a beneficial prognosis of DFS (HR = 0.56, 95% CI: 0.37–0.85) and OS (HR = 0.63, 95% CI: 0.42–0.95) for Asians, whereas it represented an adverse prognosis of DFS for Caucasians with AML (HR = 1.34, 95% CI: 1.07–1.67).

**Conclusion:**

FLT3-TKD revealed no significant effects on DFS and OS of patients with AML, which is consistent with the controversial status nowadays. Patient source (Asians or Caucasians) can be partially explained the different effects of FLT3-TKD in the prognosis of patients with AML.

## Introduction

Acute myeloid leukemia (AML) is the most common acute leukemia in adults with the features that poorly differentiated cells from hematopoietic system infiltrate in bone marrow, blood, and other tissues (1). Nowadays, it tends to be assumed that valuable and accurate prognostic assessments benefit patients with AML by providing optimized treatments for their survivals. Hence, more and more recurrent genetic mutations, such as FLT3-ITD, Nucleophosmin (NPM1), and CCAAT enhancer-binding protein alpha (CEBPA), have been used to guide disease management and refine individual prognosis.

Fms-like tyrosine kinase 3 (FLT3) is a potential prognostic genetic marker, which encodes a class 3 receptor tyrosine kinase to plays a crucial role in hematopoiesis. FLT3 gene mutations occur in approximately 30% of all patients with AML (2). There are two distinct forms of FLT3 mutations: internal tandem duplication (ITD) in the juxtamembrane domain and point mutations within the activation loop of the tyrosine kinase domain (TKD), affecting D835 in most cases (3). These gain-of-function mutations lead to ligand-independent activation of FLT3, which contributes to uncontrolled proliferation of AML blasts (2, 4, 5). Numerous studies have found that FLT3-ITD is an independent factor for adverse prognosis (6). However, the prognostic value of FLT3-TKD remains controversial due to the relatively low incidence and limitations of single center studies (2). The relationship between FLT3-TKD and cytoplasmic Src family tyrosine kinases has been confirmed recently (7) while we have known about the associations between Src family members and multiple nutrient metabolism, including glucose (8), lipid (9), and glutamine (10). From our perspectives, FLT3-TKD is regarded as a potentially metabolically related mutation in tumorigenesis and progression of AML. For this reason, we performed a meta-analysis within the published studies before 30 September 2020 to investigate the prognostic significance of FLT3-TKD in patients with AML.

## Methods

### Search strategy

Two independent investigators implemented a systematic search in PubMed, Embase, and Chinese National Knowledge Infrastructure (CNKI) databases systematically, with the last search updated on 30 September 2020. The following terms “(acute myeloid leukemia) or (acute myeloblastic leukemia) or (acute myelocytic leukemia) or (acute myelogenous leukemia) or (acute nonlymphoblastic leukemia) or AML”, “FLT3 or CD135 or (fms-like tyrosine kinase 3) or (fetal liver kinase-2) or (fetal liver kinase-3) or (human stem cell tyrosine kinase-1)”, “(tyrosine kinase domain mutation) or (TKD mutation) or D835 or I836”, and “prognosis or prognoses or (prognostic factors) or (prognostic implication) or (prognostic element)” were retrieved in PubMed entries in the National Institutes of Health and European Embase databases without any limitation applied. The key words “急性髓系白血病or急性髓性白血病or急性非淋巴细胞性白血病”, “FLT3”, and “TKD突变” were retrieved in CNKI. The reference lists in retrieved studies and relevant reviews were also manually searched for more eligible studies.

### Selection criteria

All literature studies involved in AML and FLT3-TKD were electronically retrieved for the next filter. Afterward, prospective cohort studies were identified according to their titles and abstracts. The full texts of the literature studies that fulfilled the inclusion criteria were perused to validate their eligibility. Inclusion criteria were as follows: (a) the evaluation of association between prognosis of AML and FLT3-TKD; (b) untreated patients with AML were included in study; (c) complete original materials with specific explanation of sample size; (d) they provided data of all enrolled subjects on either or both of overall survival (OS) and disease-free survival (DFS) after a period of follow-up in the study; (e) with survival information based on the FLT3 status: FLT3-TKD and wild type; and (f) prospective cohort study focusing on human being. Exclusion criteria were as follows: (a) not conforming to inclusion criteria; (b) abstract, review article, letter, comment, and editorial; (c) duplicate publication of previous publications; (d) family-based studies of pedigrees; (e) without detailed FLT3 status data (FLT3-TKD and wild type); (f) with incomplete specific explanation or without specific explanation of sample size; and (g) studies were excluded if they focused exclusively on acute promyelocytic leukemia (APL) (M3). For multiple publications from a same population, the largest study was included only to exclude duplicate studies or overlapping data. According to the inclusion and exclusion criteria, the two independent investigators accomplished study selection independently by screening title, abstract, and full text. Any dissent was solved by discussion. If agreement could not be reached, then a third researcher was consulted. Four studies were discussed and excluded after discussion (11–14).

### Data extraction

The data of the eligible studies were extracted in duplicate by two independent researchers. The data extracted comprised first author’s name, publication year, diagnostic criteria for AML, the resource of the subjects, genotyping methods, the number of subjects, the FLT3 status (FLT3-TKD or wild type) of subjects, the number of OS and/or DFS from all subjects (if any), hazard ratio (HR) with 95% confidence interval (CI) of OS and/or DFS, and the baseline data of all subjects in all included prospective cohort studies [age, sex, patient source, other mutations (if any), the usage of chemotherapeutics (if any), and so on] (**Table S1**). HR and its 95% CI were extracted directly or calculated the observed minus expected (O-E) according to the ratio of event or were extracted by using Engauge Digitizer according to the Cox curve (15). Various patient source descents were classified as Caucasian and Asian. Two investigators would check the extracted data and reach to consensus on all collected data. If a dispute existed, then original data of the included studies would be rechecked and be discussed again to reach consensus. If the dispute still existed, then the third investigators would be appointed as the decider to adjudicate the disagreements.

### Quality assessment

The Newcastle-Ottawa (NOS) scale was used to score the strength of all included studies by the three independent investigators (16). The scale has nine items classified into three major categories: selection (four items), comparability (two items), and outcome (three items) (Supplement 1. Method). In this scoring system, selection, comparability, and outcome categories could be awarded a maximum of four, two, and three points, respectively. High quality was considered as six or more points that each cohort study scored. Any discrepancies were resolved among authors. The results of quality assessment are displayed in **Table S2**.

### Publication bias and sensitivity analysis

Potential publication bias was checked by Begg’s funnel plots (17) and Egger’s test (18). An asymmetric plot with p-value less than 0.05 was considered a significant publication bias. Moreover, sensitivity analysis was performed on the pooled HRs to evaluate the effect of each study, in which the results of the meta-analysis were recalculated after removal of each study in a turn.

### Data analysis

Among entire conduction of the meta-analysis, we strictly abided by the PRISMA checklists as a guideline (19). All statistical analyses were performed with Stata 16.0 software (StataCorp, College Station, TX, USA). A two-tailed p < 0.05 was considered significant except for specified conditions, where a certain p-value was declared. HR and corresponding 95% CI were applied to assess the prognostic impact of FLT3-TKD in patients with AML. Furthermore, HR > 1 was considered as poorer prognosis in patients with FLT3-TKD than patients with FLT3-WT, whereas HR < 1 was considered as beneficial prognosis in patients with FLT3-TKD than patients with FLT3-WT. The heterogeneity of the studies was assessed by I^2^ statistic (I^2^ = 0%–25%, no heterogeneity; I^2^ = 25%–50%, moderate heterogeneity; I^2^ = 50%–75%, large heterogeneity; and I^2^ = 75%–100%, extreme heterogeneity) (20). When the heterogeneity was statistically significant (I^2^ > 50%), the random-effects model was used for assessing information; otherwise, the fixed-effects was conducted (21). Heterogeneity was analyzed by meta-regression model including age and patient source, and subgroup analysis was stratified by age and patient source. In addition, Begg’s and Egger’s tests were performed to detect potential publication bias. Furthermore, sensitivity analysis was conducted to determine the stability of findings in meta-analysis.

## Results

### Study retrieval

**Figure 1:**
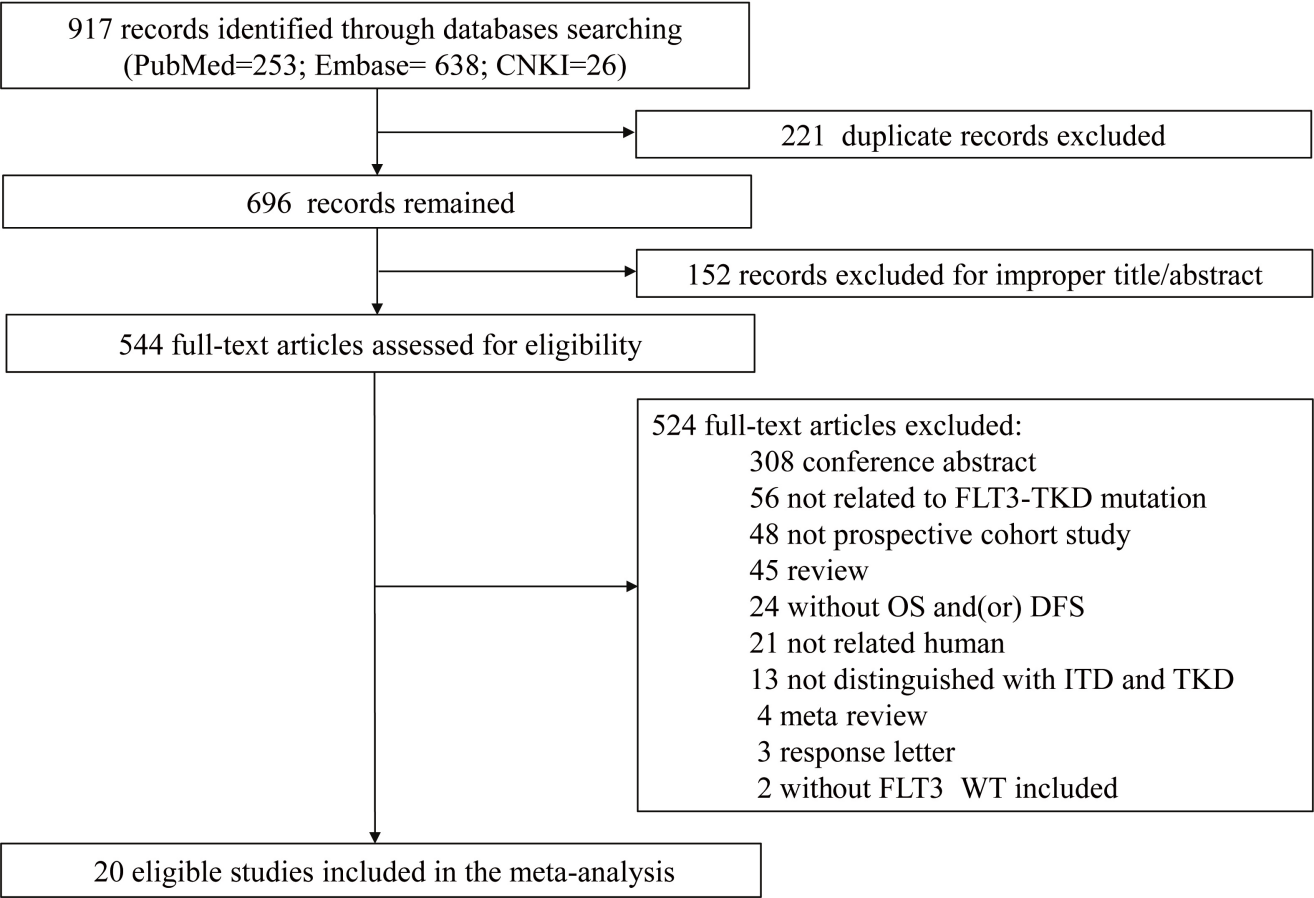
Article search and selection.

The study selection process for the meta-analysis about the prognosis of FLT3-TKD in AML is shown in **Figure 1**. Following the initial retrieval of 917 publications through database search (253 from PubMed, 638 from Embase, and 26 from CNKI), 696 relevant publications were selected after the removal of duplicates. Moreover, after a careful review of the title and abstract, 152 publications were rejected because of their irrelevance to this meta-analysis. The remaining 544 publications were full-text–reviewed; of these, 524 were excluded. The reasons for excluding are shown in **Figure 1**. Finally, 20 prospective cohort study studies (22–41) consisting of 10,970 participants (FLT3-WT = 9,744; FLT3-TKD = 1,226) were included in our meta-analysis. The general characteristics of the 20 studies are shown in **Table 1**, and additional information is shown in **Table S1**.

**Table 1:** Characteristics of studies included in the meta-analysis.

Code	First Author	Country/Region	Year	Patient number		DFS			OS		
				FLT3-WT	FLT3-TKD	HR	95% CI	p value	HR	95% CI	p value
1	Jeong Yeal Ahn	USA	2013	49	4	1.820	0.64-5.23	0.3	1.430	0.19-10.78	0.727
2	C Allen	UK	2013	319	35	NA	NA	NA	0.480	0.22-1.03	0.06
3	Costa Bachas	Netherlands	2014	123	4	0.560	0.08-4.04	0.56	0.670	0.16-2.71	0.57
4	Ulrike Bacher	Germany	2008	2935	147	1.380^*^	1.17-1.64	NA	2.010^*^	1.66-2.42	
5	Claudia Bănescu	Romania	2019	214	12	NA	NA	NA	0.900	0.47-1.70^U^	0.739
6	Prajwal Boddu	USA	2017	117	21	0.245	0.058-0.980	0.048	0.678	0.260-1.768	0.427
7	Hyoung Jin Kang	Korea	2005	55	2	0.510^#^	0.12-2.14	NA	NA	NA	NA
8	D-C Liang	Taiwan, China	2003	74	3	0.510^#^	0.16-1.66	NA	NA	NA	NA
9	Adam J Mead	UK	2007	980	127	0.710	0.52-0.97	0.03	0.710	0.52-0.96	0.03
10	Soheil Meshinchi	USA	2006	515	38	1.930^#^	1.17-3.17	NA	0.910^#^	0.57-1.45	NA
11	Isabel Moreno	Spain	2003	156	12	2.580^#^	0.63-10.52	NA	NA	NA	NA
12	Man Qiao	Mainland, China	2011	49	7	NA	NA	NA	0.429	0.204-0.653	0.779
13	Patricia Rubio	Argentina	2016	39	5	NA	NA	NA	1.410^#^	0.18-10.92	NA
14	Hirozumi Sano	Japan	2013	135	8	0.470	0.06-3.45	0.45	NA	NA	NA
15	Susanne Schnittger	Germany	2012	2676	689	1.280^*^	1.17-1.4	NA	1.350^*^	1.23-1.48	NA
16	Akira Shimada	Japan	2008	110	11	0.740^#^	0.38-1.43	NA	0.850^#^	0.45-1.58	NA
17	Christian Thiede	Germany	2002	904	75	1.750^*^	1.38-2.22	NA	1.690^*^	1.33-2.14	NA
18	Susan P Whitman	the USA	2008	123	16	2.300	1.1-4.7	0.02	NA	NA	NA
19	Y Yamamoto	Japan	2001	17	8	0.380^#^	0.18-0.8	NA	1.000^#^	0.31-3.19	NA
20	G Yoshimoto	Japan	2005	24	2	1.330^#^	0.18-10.05	NA	0.670^#^	0.15-2.9	NA

*: HR and 95% CI was by using Engauge Digitizer according to the cox curve; #: HR and 95% CI was calculated the O-E according to the ratio of event; U, univariable analysis; DFS, disease-free survival; OS, overall survival; NA, not available.

### The DFS and OS between FLT3-WT and FLT3-TKD in all patients with AML

**Figure 2:**
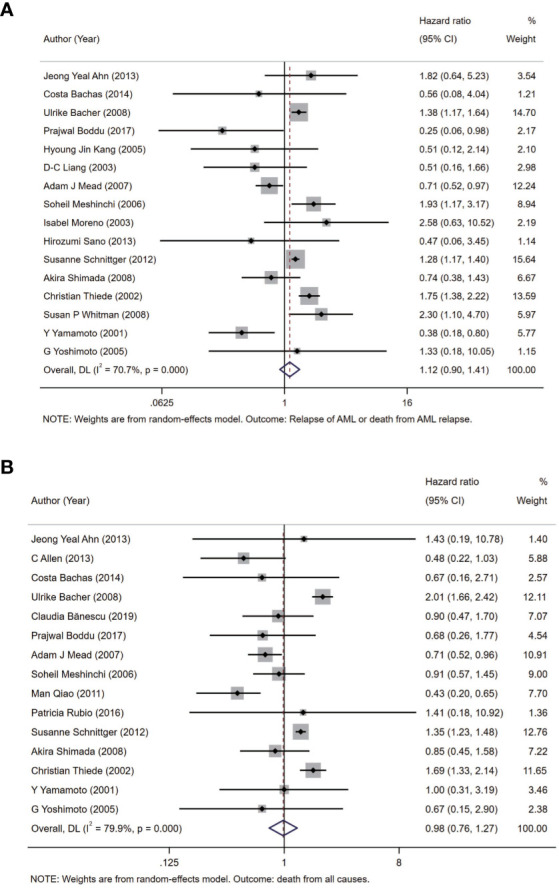
Forest plots of HRs and 95% CI for DFS **(A)** and OS **(B)** in patients with AML. The size of blocks or diamonds represents the weight in this meta-analysis, and the length of straight line segment represents the width of 95% CI. HR, hazard ratio; CI, confidence intervals; DFS, disease-free survival; OS, overall survival; AML, acute myeloid leukemia.

Before analysis, we determined two outcomes: one of them is relapse of AML or death from AML relapse as the outcome of DFS and the other one is death from all causes as the outcome of OS to represent the prognosis of AML in this meta-analysis. In all the patients with AML from included studies, we analyzed two outcomes between the group with FLT3-WT and the group with only FLT3-TKD in FLT3 gene in random-effects model. The pooled HR of DFS is 1.12 (95% CI: 0.90–1.41; I^2^ = 70.7%, p = 0.000) (**Figure 2A**). In same model, the pooled HR of OS is 0.98 (95% CI: 0.76–1.27; I^2^ = 79.9%, p = 0.000) (**Figure 2B**).

### Meta-regression was used for analyzing heterogeneity

**Figure 3:**
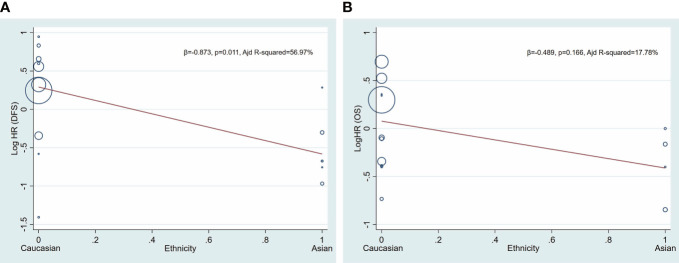
Univariate meta−regression analysis. Log HR of DFS and OS according patient source (ethnicity). A circle represents a study, and the diameter of circle represents sample capacity. Various ethnicity (patient source) descents were classified as Caucasian and Asian. HR, hazard ratio; DFS, disease-free survival; OS, overall survival.

Age and patient source were included in meta-regression model for heterogeneity analysis. The coefficient of age is −0.005 (p = 0.497) and 0.008 (p = 0.299) in patients of AML for DFS and OS, respectively, and the coefficient of patient source is −1.004 (p = 0.015) and −0.358 (p = 0.324) in patients of AML for DFS and OS, respectively (**Table 2**, **Figure 3**).

**Table 2:** Multivariate meta-regression analysis for FLT3-TKD in patients of AML.

Outcome	DFS		OS	
	Coefficient	p-value	Coefficient	p-value
Age	−0.005	0.497	0.008	0.299
Patient source	−1.004	0.015*	−0.358	0.324

DFS, disease-free survival; OS, overall survival; *p ＜ 0.05.

### The DFS and OS of patients with AML between FLT3-WT and FLT3-TKD in the Asian subgroup and in the Caucasian subgroup

**Figure 4:**
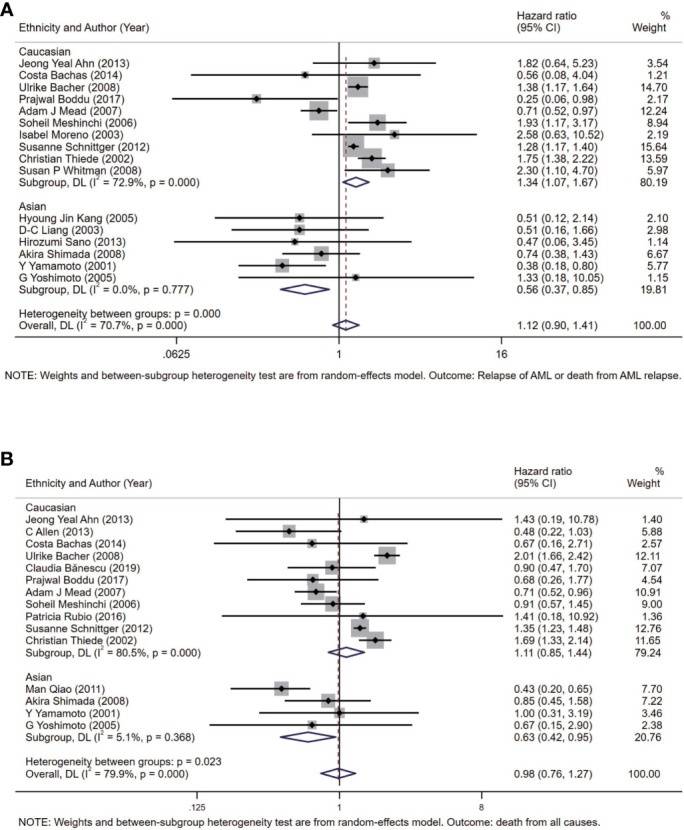
Forest plots of HRs and 95% CI for DFS **(A)** and OS **(B)** in patients with AML in the Asian subgroup and the Caucasian subgroup. The size of blocks or diamonds represents the weight in this meta-analysis, and the length of straight line segment represents the width of 95% CI. HR, hazard ratio; CI, confidence intervals; DFS, disease-free survival; OS, overall survival; AML, acute myeloid leukemia.

To further analyze heterogeneity, considering with the background knowledge of FLT3-TKD, we conducted meta-analysis in subgroups according to the patient source (ethnicity) of all participants included (**Table S1**). The pooled HR of DFS is 1.34 (95% CI: 1.07–1.67; I^2^ = = 72.9%, p = 0.000) in the Caucasian subgroup, and the pooled HR of DFS is 0.56 (95% CI: 0.37–0.85; I^2^ = 0.0%, p = 0.777) in the Asian subgroup (**Figure 4A**). The pooled HR of OS is 1.11 (95% CI: 0.85–1.44; I^2^ = 80.5%, p = 0.000) in the Caucasian subgroup, and the pooled HR of OS is 0.63 (95% CI: 0.42–0.95; I^2^ = 5.1%, p = 0.368) in the Asian subgroup (**Figure 4B**).

### Publication bias and sensitivity analysis

**Figure 5:**
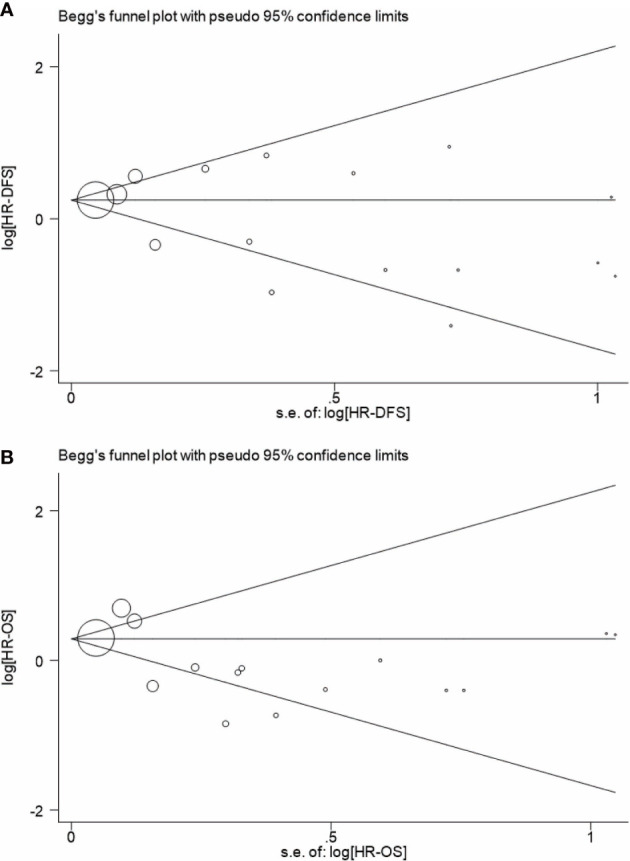
Begg’s funnel plot for analyzing publication bias of DFS **(A)** and OS **(B)** in patients with AML. A circle represents a study, and the diameter of circle represents sample capacity. DFS, disease-free survival; OS, overall survival; AML, acute myeloid leukemia.

**Figure 6:**
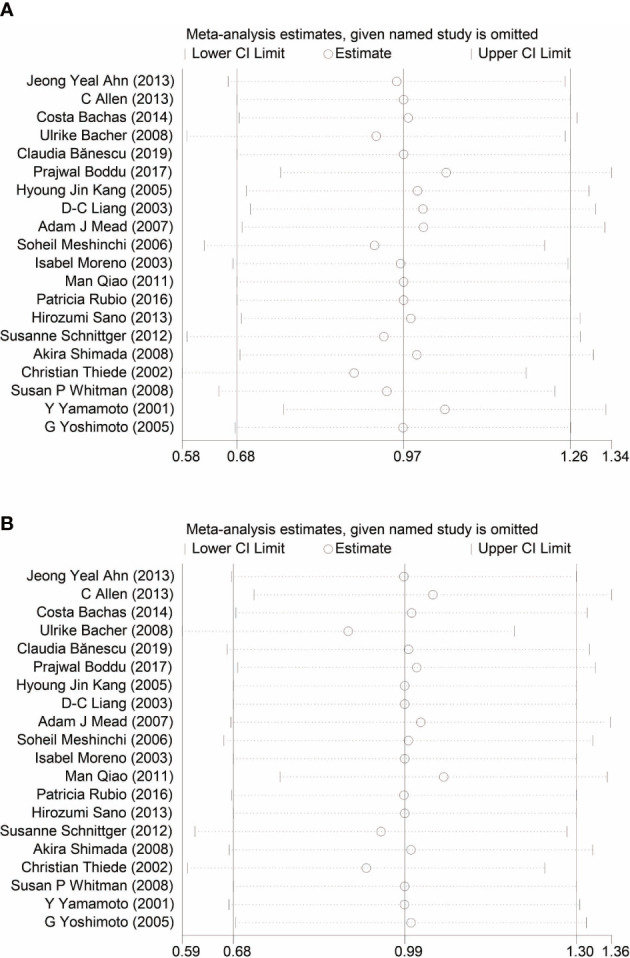
Sensitivity analysis of DFS **(A)** and OS **(B)** in patients with AML. DFS, disease-free survival; OS, overall survival; AML, acute myeloid leukemia.

Begg’s and Egger’s tests have been used to detect any publication bias that indicated that there was no significant bias between studies of FLT3-TKD in prediction of DFS (p = 0.499 of Begg’s and p = 0.233 of Egger’s) and also of OS (p = 0.488 of Begg’s and p = 0.061 of Egger’s). Symmetrical Begg’s funnel plot was obtained (**Figure 5**). We also conducted sensitivity analysis to examine the stability of the meta-analysis to determine the influence of each study on pooled HRs in patients with AML by deleting a single study in every model. It showed no individual study affected the pooled HR of DFS (**Figure 6A**) and OS (**Figure 6B**) in all patients with AML.

## Discussion

In this meta-analysis, 20 prospective cohort studies (n = 10,970) on FLT3-TKD in AML were included: 9,744 subjects with FLT3-WT and 1,226 subjects with FLT3-TKD (22–41). The incidence of FLT3-TKD in this meta-analysis was 11.2%, which was nearly consistent with previous studies (approximately 7%–10% of all AML cases) (2). Our results indicated that, within 20 cohort studies (n = 10,970) included, the pooled HR of DFS was 1.12 (95% CI: 0.90–1.41; I^2^ = 70.7%, p = 0.000) and OS was 0.98 (95% CI: 0.76–1.27; I^2^ = 79.9%, p = 0.000), which revealed no significant effect of FLT3-TKD on DFS and OS of patients with AML by random effect models. However, meta-regressions demonstrated that patient source associated with the prognosis effect of FLT3-TKD in patients with AML. To be specific, FLT3-TKD represented a beneficial prognosis of DFS (HR = 0.56, 95% CI: 0.37–0.85) and OS (HR = 0.63, 95% CI: 0.42–0.95) for Asians with AML, whereas FLT3-TKD represented an adverse prognosis of DFS for Caucasians with AML (HR = 1.34, 95% CI: 1.07–1.67). However, the results of DFS from Caucasians ought to be interpreted with caution due to the heterogeneity (I^2^ = 72.9%, p = 0.000).

In general, FLT3-TKD reveals no significant effects on DFS and OS of patients with AML, which is consistent with the controversial statue nowadays. The controversies of prognostic impacts on FLT3-TKD in patients with AML were supported by two-sided laboratory evidence of FLT3-TKD. On one hand, many studies indicated that FLT3-TKD associated a beneficial prognosis of AML. In some cases, patients with FLT3-TKD companying with other mutations, such as NPM1 mutation, showed a favorable prognosis (27) with the reasons that the localization and signaling of FLT3-TKD was changed by NPM1c in AML (42). On the other hand, other research studies manifested that FLT3-TKD was considered as a harmful mutation in prognosis of AML. Since FLT3-ITD was first recognized as a frequently mutated gene in AML in 1996 (43), growing studies indicated that a lot of FLT3-ITD–positive patients with AML relapse with the appearance of FLT3-TKD after initial response to FLT3 inhibitor treatments. Moreover, several FLT3 inhibitors including Sorafenib and Quizartinib potently had effects on inhibiting FLT3-ITD but were not effective toward FLT3-TKD (2, 44, 45). The phenomenon was plausibly interpreted as the coexistence of two kinds of FLT3 mutations and the presence of FLT3-TKD in a very low level at initial stage of disease, which subsequently became prevalent after FLT3-ITD–positive leukemic cells, are eliminated (46).

Our results indicated that FLT3-TKD represented a beneficial prognosis for Asians with AML, whereas it represented an adverse prognosis of DFS for Caucasians with AML, but with heterogeneity. First, in the Caucasian subgroup, the pooled HR of DFS was 1.34 (95% CI: 1.07–1.67; I^2^ = 72.9%, p = 0.000), and the pooled HR of OS was 1.11 (95% CI: 0.85–1.44; I^2^ = 80.5%, p = 0.000) (**Figure 4**). There were 635 Asians from four different countries and 10,335 Caucasians from eight different countries in this meta-analysis, which revealed that Caucasians outnumbered Asians. Hence, we speculated that confounding factors from large sample capacity of the Caucasian subgroup accounted for the heterogeneity in Caucasians (I^2^ = 72.9%, p = 0.000). In this situation, if related confounding factors were well controlled, then the conclusion might be more convinced. However, it was indicated that Caucasians reveals a distinct genetic alteration profiles of AML than Eastern Asian population (47), but what we focused on is the ratio of FLT3-TKD in AML, which would not be influence by population base too much at this meta-analysis. Second, according to different therapeutic guidelines of AML, the chemotherapy regimens for patients with AML vary internationally. On one hand, included studies from Japan accounted for a large population in the Asian subgroup in this meta-analysis. The conventional “3 + 7” induction is regarded as basic regimens for complete remission (CR) with anthracyclines for 3 days and standard dose of cytarabine for 7 days. We noticed that the dose of cytarabine and anthracyclines in Japan was less than the dose in Caucasian countries according to practical guideline for AML (48, 49). Cytarabine of 100 mg/m^2^ was recommended for 7 days in Japan, whereas cytarabine of 100–200 mg/m^2^ was recommended for 7 days in Caucasian countries; and daunorubicin of 50 mg/m^2^ was recommended for 3 days in Japan, whereas daunorubicin of 60–90 mg/m^2^ for 3 days was recommended in Caucasian countries (48, 49). Hence, we speculated prognosis of AML in different countries or regions might attribute to the dose of cytarabine and daunorubicin, which consist of the conventional “3 + 7” induction. On another hand, anthracyclines have been used extensively with standard dose of cytarabine to induce remission of patients with AML worldwide (48, 50). However, clinicians in China tend to attach great importance to homoharringtonine (HHT) and apply HHT-based induction regimens to induce the CR of patients with AML (not APL), which was considered as another discrepancy between Asian countries and Caucasian countries in the treatments of AML (48, 50). Concretely, clinical hematologists and oncologists in China are apt to replace anthracyclines with HHT in CR induction of AML or added HHT upon prime chemotherapy regimens for higher CR rate of AML. HHT is a kind of alkaloid deriving from genus Cephalotaxus and exerts selective antileukemia effects on patients with AML, especially on these carrying FLT3-ITD and elderly patients with AML (51–53). What is noteworthy is that the FLT3-TKD status associates with chemotherapy regimens, especially in relapse (2), whereas some salvage therapies for relapsed AML include HHT as the basic members of chemotherapy regimens, such as HAA regimen (54). Hence, we speculated that prognosis of AML in different ethnicities may also due to the clinical usage of HHT partially. Third, race diversity is the result of different genetic backgrounds, not only the gene encoding FLT3. Patients with AML usually were with genomic anomaly or sporadic gene mutations (42). Currently, the contribution of different genetic backgrounds to the occurrence and progression of AML remains unclear. In conclusion, FLT3-TKD exerts impacts on contrasting prognosis of AML in different ethnicities due to multiple reasons, which deserve further explorations.

FLT3-TKD is considered as a potentially metabolically related mutation in AML. FLT3-TKD associates with cytoplasmic Src family tyrosine kinases by increasing the phosphorylation of activating tyrosines, such as FGR and HCK (7). In addition, Src family members are involve in multiple nutrients metabolisms, including glucose (8), lipid (9), and glutamine (10), indicating that FLT3-TKD is a potentially metabolically related mutation. As for glucose metabolism, Src is able to regulate cyclin B1, N-cadherin, and E-cadherin under high glucose as a response to hyperglycemia in colorectal cancer (8). For glutamine metabolism, epidermal growth factor receptor (EGFR)-promoted tumor progression is considered as being Src signaling pathway related by influencing glutamine metabolism in multiple malignancies (10). For lipid metabolism, Src is able to being recruited by CDCP1 into lipid rafts, which affect HGF receptor Met *via* the activation of STAT3 (9). Overall, FLT3-TKD is considered as being metabolically related based on the functions of Src family tyrosine kinases, which can be used to explain FLT3-TKD status in AML.

Our meta-analysis has several strengths. First, we selected 20 eligible prospective cohort studies, which was considered as an ideal epidemiological method to predict prognosis of AML. In addition, selection bias was well controlled by two independent investigator and an unlimited literature search. Furthermore, most included studies were of high quality with regard to quality assessment of the NOS scale (16). Moreover, no evident publication bias was identified by either Begg’s funnel plot or Egger’s test. Finally, we conducted sensitivity analysis by deleting a single study in every model, and we did not find obvious abnormal studies contributing to the pooled HR.

Begg’s funnel plot was used to detect publication bias in this meta-analysis (**Figure 5**): As is shown in **Figure 5**, publication bias is not evident when DFS was regarded as an evaluated end point (**Figure 5A**); however, it indicated a publication bias when OS was regarded as an evaluated end point (**Figure 5B**). Indeed, OS reflects the multiple influences to individuals, not just AML. The OS of AML is able to attribute to multiple factors, which make the manuscript easy to publish. Moreover, OS can be concluded according to the status of the patients with AML (live/dead), whereas DFS is usually diagnosed on the basis of medical examination, such as bone marrow biopsy. The difference contributed to the difficulty of obtaining data from AML, which also may lead to the publication bias. All in all, DFS of AML is considered as a better evaluated index in the prognosis AML, and DFS of AML in this meta-analysis did not indicate a publication bias.

There are several limitations of this study that should be acknowledged. First, there was a heterogeneity in Caucasians (I^2^ = 72.9%, p = 0.000), which may attribute to the fact that Caucasians outnumbered Asians: 635 Asians from four different countries and 10,335 Caucasians from eight different countries in this meta-analysis. In our opinion, the heterogeneity from Caucasian group was from some unknown confounding factors in the united prospective cohorts of Caucasians. In this situation, if related confounding factors were well controlled, then the conclusion might be more convinced. In addition, we failed to get the specific information about chemotherapy from 10,970 participants including chemotherapy regimens, chemotherapy dose, and chemotherapy time, because different types of treatment may exert distinct impacts on the prognosis of the patients with AML. However, FLT3-TKD does not result from general chemotherapies, so the FLT3-TKD in AML in this study is regarded as being from individuals’ genetic backgrounds. Furthermore, because of the limitation from the extracted data, we were unable to perform more stratification analysis according to other confounding factors. Although confounding factors work on the ending events, it is considered as a common problem in clinical research studies because we cannot predict everything before the start.

In conclusion, our results showed that FLT3-TKD revealed no significant effect on DFS and OS of patients with AML. However, meta-regressions demonstrated that patient source associated with the prognosis effect of FLT3-TKD in patients with AML. To be specific, FLT3-TKD represented a beneficial prognosis of DFS and OS for Asians with AML, whereas FLT3-TKD represented an adverse prognosis of DFS for Caucasians with AML. However, the results of DFS from Caucasians ought to be interpreted with caution due to the heterogeneity. This meta-analysis provided new information about the distinct prognosis of patients with AML between Asians and Caucasians. From our perspectives, the Caucasians with FLT3-TKD at the initial diagnostic stage of AML could be recommended the Asians dose of cytarabine and daunorubicin (cytarabine of 100 mg/m^2^ + daunorubicin of 50 mg/m^2^) in conventional “3 + 7” induction, so that they could receive a better prognosis of AML for survivals. An adequately designed prospective study including a large population with AML with clear FLT3 gene statue is needed to confirm our results.

## Role of the funding source

The funder of the study supported the study design, data analysis, and publication of the manuscript.

## Data availability statement

The original contributions presented in the study are included in the article/**Supplementary Material**. Further inquiries can be directed to the corresponding authors.

## Author contributions

SL was involved in project conceiving, data collection, data curation, literature assessment, investigation, formal analysis, software performance, visualization, and original manuscript writing. NL was involved in data collection, data curation, and literature assessment. YC was involved in project conceiving, study supervision, and manuscript editing. ZZ was involved in study supervision and funding acquisition. YG was involved in project conceiving, literature assessment, study supervision, investigation, funding acquisition, and manuscript editing. All of authors read and approved the final manuscript.
