# Supplementary material for: FLT3-TKD in the prognosis of patients with acute myeloid leukemia: A meta-analysis
Source: Front Oncol. 2023 Feb 17;13:1086846. doi: 10.3389/fonc.2023.1086846 (PMC9982020; doi:10.3389/fonc.2023.1086846)
Supplement: Supplementary file 1 [file DataSheet_1.docx]

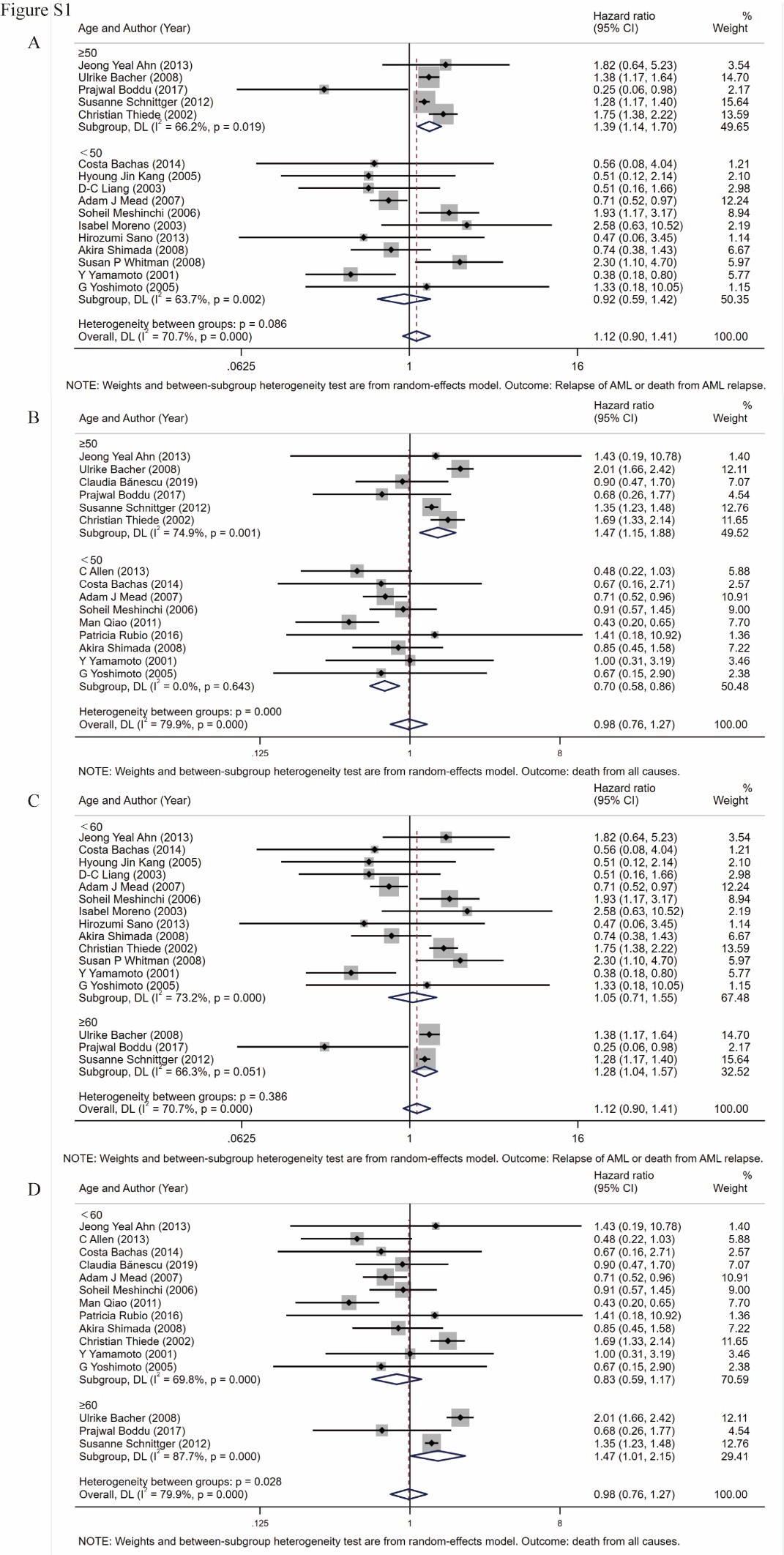
**Supplementary Material**

**Figure Legend**

**Figure S1:** Forest plots of the HRs and 95% CI for DFS (A&C) and OS (B&D) in patients with AML in multiple age subgroup. The size of the blocks or diamonds represents the weight and the length of the straight line represents the width of 95% CI. HR, hazard ratio; CI, confidence intervals; DFS, disease free survival; OS, overall survival; AML, acute myeloid leukemia.

| **Table S1 Additional information of studies included in the meta-analysis.** | | | | | | | | | | | | | |
| --- | --- | --- | --- | --- | --- | --- | --- | --- | --- | --- | --- | --- | --- |
| **Code** | **Author** | **Country** | **Year** | **NO.** | |  | **Age** | | | | | | **Patient source** |
|  |  |  |  | **WT** | **TKD** | | **WT** | | **TKD** | | | **Median** |  |
| 1 | Jeong Yeal Ahn | the USA | 2013- | 49 | 4 | | 23 patients are Younger than 60 (43.4%)  30 patients are 60 and older (56.6%) | | | | | 52.6 | Caucasian |
| 2 | C Allen | UK | 2013 | 319 | 35 | | 39 (15-70) | | 38 (30-63) | | | 39 | Caucasian |
| 3 | Costa Bachas | Netherlands | 2014 | 123 | 4 | | 10.2 (0.4-19.5) | | | | | 10.2 | Caucasian |
| 4 | Ulrike Bacher | Germany | 2008 | 2935 | 147 | | 63.1 (17.5-91.8) | | | | | 63.1 | Caucasian |
| 5 | Claudia Bănescu | Romania | 2019 | 214 | 12 | | 54.44 ± 16.78* (range 19-87 years old) | | | | | 54.4 | Caucasian |
| 6 | Prajwal Boddu | the USA | 2017 | 117 | 21 | | 62 (17-88) | | | | | 62 | Caucasian |
|  |  |  |  |  |  |  | 63 (17-88) | | | 58 (28-84) | |  |  |
| 7 | Hyoung Jin Kang | Korea | 2005 | 55 | 2 | | 7 years 9 months (4 months-17 years 2 months) | | | | | 7.75 | Asian |
| 8 | D-C Liang | Taiwan | 2003 | 74 | 3 | | ≤18 | | 13/3/6# | | | ≤18 | Asian |
| 9 | Adam J Mead | UK | 2007 | 980 | 127 | | 42 | | 43 | | | 42 | Caucasian |
| 10 | Soheil Meshinchi | the USA | 2006 | 515 | 38 | | 10.0 (0.01-20.9) | | 11.0 (0.3-19.7) | | | 10.1 | Caucasian |
| 11 | Isabel Moreno | Spain | 2003 | 156 | 12 | | ≤ 20 | 14.3% | ≤ 20 | | 10% | 45 | Caucasian |
|  |  |  |  |  |  |  | 21-50 | 31% | 21-50 | | 45% |  |  |
|  |  |  |  |  |  |  | > 50 | 54.8% | > 50 | | 45% |  |  |
| 12 | Man Qiao | China | 2011 | 49 | 7 | | 37.5(13-76) | | | | | 37.5 | Asian |
| 13 | Patricia Rubio | Argentina | 2016 | 39 | 5 | | 7.0 years (range 1 day–17.9 years) | | | | | 7 | Caucasian |
|  |  |  |  |  |  |  | 4.0 (0-11.0) | | 6.0 (4.0-7.0) | | |  |  |
| 14 | Hirozumi Sano | Japan | 2013 | 135 | 8 | | 6 (0-15) | | | | | 6 | Asian |
|  |  |  |  |  |  |  | 6 (0-15) | |  | | |  |  |
| 15 | Susanne Schnittger | Germany | 2012 | 2676 | 689 | | 63.2 (15.8-91.8) | | | | | 63.2 | Caucasian |
|  |  |  |  |  |  |  | 63.6 (15.8-91.8) | | 60.4 (16.3-89.2) | | |  |  |
| 16 | Akira Shimada | Japan | 2008 | 110 | 11 | | 6 (0-15) | | | | | 6 | Asian |
|  |  |  |  |  |  |  |  | | 11 (2-14) | | |  |  |
| 17 | Christian Thiede | Germany | 2002 | 904 | 75 | | ＜20  21-30  31-40  41-50  51-60  61-70  ＞70 | | 27  64  126  155  238  289  114 | | | 52 | Caucasian |
| 18 | Susan P Whitman | the USA | 2008 | 123 | 16 | | 47 (19-59) | | 43 (19-57) | | | 46.5 | Caucasian |
| 19 | Y Yamamoto | Japan | 2001 | 147 | 8 | | 49 (15-85) | | | | | 49 | Asian |
|  |  |  |  |  |  |  | 48 (15-85) | | 53.5 (17-70) | | |  |  |
| 20 | G Yoshimoto | Japan | 2005 | 24 | 2 | | 41.5 (15-74) | | | | | 41.5 | Asian |
|  |  |  |  |  |  |  | 42 (17-74) | | 28 (21-35) | | |  |  |

Age was presented as the median (95%CI)

Ethnicity was presented as Caucasian and Asian

*represent as mean age ± SD

#the age of 3 AML patients respectively

| **Table S2 Newcastle-Ottawa risk of bias scores for the twenty studies included in this meta-analysis.** | | | | | | | | | |
| --- | --- | --- | --- | --- | --- | --- | --- | --- | --- |
| **First author, year** | **Selection** | | | | **Comparability** | **Outcome** | | | **Total** |
|  | **Representativeness** | **Non-Exposed** | **Exposure** | **Outcome** |  | **Assessment of Outcome** | **Follow-Up Long** | **Adequacy of Follow Up** |  |
| Jeong Yeal Ahn, 2013 | 0 | 1 | 1 | 1 | 0 | 1 | 1 | 1 | 6 |
| C Allen, 2013 | 1 | 1 | 1 | 1 | 0 | 1 | 1 | 1 | 7 |
| Costa Bachas, 2014 | 0 | 1 | 1 | 1 | 0 | 1 | 0 | 1 | 5 |
| Ulrike Bacher, 2008 | 1 | 1 | 1 | 1 | 0 | 1 | 1 | 1 | 7 |
| Claudia Bănescu, 2019 | 1 | 1 | 1 | 1 | 0 | 1 | 0 | 1 | 6 |
| Prajwal Boddu, 2017 | 1 | 1 | 1 | 1 | 2 | 1 | 1 | 1 | 9 |
| Hyoung Jin Kang, 2005 | 0 | 1 | 1 | 1 | 0 | 1 | 1 | 1 | 6 |
| D-C Liang, 2003 | 0 | 1 | 1 | 1 | 0 | 1 | 0 | 1 | 5 |
| Adam J Mead, 2007 | 1 | 1 | 1 | 1 | 2 | 1 | 1 | 1 | 9 |
| Soheil Meshinchi, 2006 | 1 | 1 | 1 | 1 | 0 | 1 | 1 | 1 | 7 |
| Isabel Moreno, 2003 | 1 | 1 | 1 | 1 | 0 | 1 | 1 | 1 | 7 |
| Man Qiao, 2011 | 0 | 1 | 1 | 1 | 0 | 1 | 1 | 1 | 6 |
| Patricia Rubio, 2016 | 0 | 1 | 1 | 1 | 0 | 1 | 1 | 1 | 6 |
| Hirozumi Sano, 2013 | 0 | 1 | 1 | 1 | 1 | 1 | 1 | 1 | 7 |
| SusanneSchnittger, 2012 | 1 | 1 | 1 | 1 | 2 | 1 | 1 | 1 | 9 |
| Akira Shimada, 2008 | 1 | 1 | 1 | 1 | 0 | 1 | 1 | 1 | 7 |
| Christian Thiede, 2002 | 1 | 1 | 1 | 1 | 0 | 1 | 1 | 1 | 7 |
| Susan P Whitman, 2008 | 1 | 1 | 1 | 1 | 1 | 1 | 1 | 1 | 8 |
| Y Yamamoto, 2001 | 0 | 1 | 1 | 1 | 0 | 1 | 1 | 1 | 6 |
| G Yoshimoto, 2005 | 0 | 1 | 1 | 1 | 0 | 1 | 1 | 1 | 6 |

**Supplement 1. Method.**

**CODING MANUAL FOR COHORT STUDIES**

# SELECTION

1. **Representativeness of the Exposed Cohort**

Item is assessing the representativeness of individuals with mere FLT3-TKD mutation in FLT3 gene and with a definite AML diagnosis.

1 point: Subjects were with well representativeness.

0 point: Subjects were with poor representative, such as unclear FLT3 status, unclear AML diagnosis and so on.

Allocation of stars as per rating sheet.

1. **Selection of the Non-Exposed Cohort**

1 point: Subjects were detected with appropriate methods for FLT3-TKD mutation.

0 point: Subjects were detected with defective methods for FLT3-TKD mutation or detecting methods are unreliable.

Allocation of stars as per rating sheet.

1. **Ascertainment of Exposure**

1 point: Subjects were detected with appropriate methods for FLT3-TKD mutation.

0 point: Subjects were detected with defective methods for FLT3-TKD mutation or detecting methods are unreliable.

Allocation of stars as per rating sheet.

1. **Demonstration That Outcome of Interest Was Not Present at Start of Study**

In the included studies, outcome of interest is still the survival state of patients with AML to reflect the prognosis. That is to say that a clear statement of free survival and/or overall survival earns a point.

Allocation of stars as per rating sheet.

***COMPARABILITY***

1. **Comparability of Cohorts on the Basis of the Design or Analysis**

A maximum of 2 points can be allotted in this category.

Either exposed (with FLT3-TKD mutation) and non-exposed (with FLT3-WT) individuals must be matched in the design and/or confounders must be adjusted for in the analysis. Statements of no differences between groups or that differences were not statistically significant are not sufficient for establishing comparability. Note: If the relative risk for the exposure (with FLT3-TKD mutation) of interest is adjusted for the confounders listed, then the groups will be considered to be comparable on each variable used in the adjustment. There may be multiple ratings for this item for included adjusting factors: Age earns 1 point and other controlled factors earns 1 point.

Allocation of stars as per rating sheet

***OUTCOME***

1. **Assessment of Outcome**

We used the hazard ratio (HR) to represent outcomes [disease free survival (DFS) or overall survival(OS)] in patients with AML.

1 point: The value of HR and 95% CI were extracted by direct extraction, or by calculated the O-E according to the ratio, or extraction by using Engauge Digitizer according to the cox curve.

0 point: No description or no data was available.

Allocation of stars as per rating sheet

1. **Was Follow-Up Long Enough for Outcomes to Occur**

An acceptable length of time should be decided before quality assessment begins (e.g. 1 year.)

Allocation of stars as per rating sheet.

1. **Adequacy of Follow Up of Cohorts**

This item assesses the follow-up of the exposed (with FLT3-TKD mutation) and non-exposed (with FLT3-WT) cohorts to ensure that losses are not related to the outcome.

Allocation of stars as per rating sheet.
